# Social Support, Religious Endorsement, and Career Commitment: A Study on Saudi Nurses

**DOI:** 10.3390/bs8010008

**Published:** 2018-01-10

**Authors:** Mohammad T. Azim, Mohammad M. Islam

**Affiliations:** 1 Department of Business Administration, King Abdulaziz University, Jeddah 21589, Saudi Arabia; 2 Department of Finance, College of Business, King Abdulaziz Univerity, Jeddah 21589, Saudi Arabia; mazharulislam@yahoo.com

**Keywords:** career commitment, nurse, religious endorsement, Saudi Arabia, social support

## Abstract

The present study investigates the effect of perceived social support (PSS) and perceived religious endorsement (PRE) on career commitment (CC) of Saudi nurses. The investigation also extends to the moderating role of different demographic and organizational factors in the extent of PSS, and career commitment these nurses report. Data required for meeting these study objectives were collected from male and female Saudi nurses through a structured questionnaire. Multiple regressions using Partial Least Squares based Structural Equation Model, Smart-PLS version 3.0, and independent sample *t*-test using SPSS version 22.0, were used to analyze data. The study findings reveal that both perceived social support and perceived religious endorsement are important antecedents of career commitment of Saudi nurses. However, private-sector nurses are found to exhibit a significantly higher level of career commitment compared to their public-sector counterparts. Nurses with greater educational attainment perceive higher level of social support and express greater career commitment than their less educated peers. These findings suggest that nursing as a profession should be more openly discussed in both secular and religious contexts, to ensure an adequate level of respect and compassion on behalf of the public. In particular, endorsement from the individual nurses’ social networks is vital in maintaining their wellbeing and career commitment. Given the religious influence in all aspects of life in the Saudi society, the current practice of gender-based segregation in Saudi hospitals and clinics seems to be meaningful for sustaining the career commitment of the nurses.

## 1. Introduction

While the shortage of nursing professionals is a worldwide phenomenon owing to the rapidly growing population, it is of particular concern in Saudi Arabia due to the adverse societal attitudes toward this profession. According to the World Health Organization [1], at 48.7 nursing and midwife professionals per 10,000 persons, Saudi Arabia is far below the 88.2 reported for high-income countries. The societal perceptions of the profession and cultural factors are largely attributed to this shortage [2]. Indeed, empirical evidence indicates that nursing is not considered a respectable profession in Saudi Arabia, particularly for women [2]. Similarly, in a study on Saudi high school students’ perceptions of nursing as a possible career, Al-Omar [3] concluded, “community image, family disagreement, cultural and communal values, long working hours, night shifts, interaction with members of the opposite gender, and the worry of not being a ‘marriageable’ prospect were the main reasons for not choosing nursing as a career by Saudi women” (p. 151). Consequently, Saudi Arabia, like other Middle Eastern countries, has been heavily dependent on expatriates to make up the shortfalls in the healthcare sector. However, over the years, the number of Saudi nationals pursuing nursing as an educational and career choice has been increasing gradually, and they now constitute around 36% of the total nursing workforce in the country [4]. On the contrary, the alarming concern regarding the profession is its high turnover rate, which is as high as 30% [5]. This finding, to some extent, questions the career commitment of Saudi nurses. Empirical evidence indicates that, due to the cultural and personal factors, commitment to the career, as well as the level of professionalism among Saudi nurses mostly depends upon the social support they receive from their family and friends. Moreover, life in Saudi Arabia is strongly influenced by Islamic beliefs and principles. Islamic scholars consider it disgraceful for Muslim women to work outside the home. Most importantly, intermingling of men and women in the workplace is prohibited in Islam [2]. Thus, religious beliefs also affect career commitment of Saudi nurses. Given these factors and the need to increase participation of Saudi nurses in the local workforce, the aim of the present study is to determine the effect of perceived social support (PSS) and perceived religious endorsement (PRE) on career commitment (CC) of Saudi nurses. It also intends to observe the moderating effects of a few selected demographic and organizational factors like, age, gender, marital status, education, experience, and the type of organization on PSS and CC of Saudi Nurses.

## 2. Nursing in Saudi Arabia

Nursing is a relatively new profession to the Saudi society. Prior to 1960, healthcare in Saudi Arabia primarily relied on traditional forms of treatment and care. There were no nursing, or medical schools and Saudi people had not participated in the nursing profession [6]. Even after the establishment of health services, the country has relied heavily on expatriate nurses, recruited from more than 40 countries that has impeded the professional growth of Saudi nurses [6]. Miller-Rosser et al. [2] provided a brief chronology of nursing education in the country, along with the historical roots and socio-cultural factors affecting the profession in the Saudi Arabia. They concluded that “Nursing is still experiencing mixed reactions that truncate its development and diminish its appeal to young Saudi women. Nevertheless, in spite of continuing negativism, the number of Saudi women entering the profession is increasing. As these women become proficient in clinical skills and critical thinking, their awareness of the barriers that impede the development of their chosen profession is increased”. 

Al-Omar [3] examined the knowledge, attitudes, and intention among the Saudi high school students toward the nursing profession, reporting that most of the participants had a negative attitude toward the profession, while only 5.2% of the students indicated that they would consider nursing as a career option. These findings are supported by the results reported by Al-Malki, FitzGerald and Clark [7], who cited a constant shortage of staff, as well as a high rate of turnover among the Saudi nurses. More recently, Alboliteeh [8] in his doctoral research revealed that Saudi nurses suffered from poor social status associated with the profession and were subject to criticism and poor treatment by family, other health professionals, and society at large. However, he also reported that the nurses, in general, had a positive attitude to their profession. In his inquiry about the intention to leave the profession, approximately 43% of respondents admitted that they had the plan to quit the profession at some point in the future. The most prominent reasons to quit the profession identified by the study were long working hours (86%) and dealing with the opposite sex (50%). 

As nursing is not traditionally a popular career choice in Saudi Arabia, the choice and quality of nursing education progressed over time. Initially, a one-year certificate course on nursing was offered, with the graduates employed as assistant nurses. Currently, nursing education takes place under the initiatives of three types of institutes, namely, health institutes, junior colleges, and universities. Health institutes and junior colleges offer three-year Diploma in Nursing, while the universities offer Bachelor of Science in Nursing (BSN) and Masters of Science in Nursing degrees. Nurses who graduate from health institutes and junior colleges and hold diplomas are classified as technical nurses and senior technical nurses, respectively. Nurses that hold a Bachelor of Nursing qualification are classified as specialists, while nurses that have completed a Masters of Nursing Science postgraduate degree are classified as senior specialists [9]. Even though nursing is regarded as a female profession globally, given that in Saudi Arabia women are discouraged from working outside the home, there is an almost equal ratio of places in the aforementioned educational institutions designated for male and female students. The reason for providing equal opportunities for all students wishing to pursue nursing as a career likely stems from the need to ensure that there is a sufficient number of female nurses to attend to female patients. This segregation of patient care in line with gender is promoted by the government to serve the cultural needs of the population. 

## 3. Perceived Social Support

Perceived social support refers to an individual’s cognitive appraisal of support a person receives from his/her social network. The support may emerge in three forms: (a) emotional (e.g., empathy, caring, love, and trust); (b) informational (e.g., advice, suggestions, access to information, etc.); and (c) instrumental (e.g., sharing of tasks and responsibilities, aid in kind, skills acquisition, etc.) [10]. Authors of extant studies on perceived social support found it to be a strong antecedent of reduced stress levels and lower prevalence of psychological disorders, as well as higher perceived sense of wellbeing [11,12]. Social support acts as a buffer and protects the recipients from the detrimental effects of stressful events, which, in turn, improves their health and wellbeing. Empirical evidence also suggests that perceived social support has a similar impact on reducing stress and improving wellbeing as does the actual support [11,13,14].

Since the nursing profession usually demands a high level of personal commitment, due to long working hours, direct contact with patients and their families, the need to deal with challenging cases and delicate people, and night shifts, it is usually viewed as both physically and mentally stressful and emotionally difficult job. In the context of Saudi Arabia, because of the poor image of the profession, along with social and religious stigma associated with nursing, the job is even more stressful than in other countries. Therefore, career commitment as well as the professional excellence of nurses in Saudi Arabia is likely to be largely dependent on the support they receive from their social networks, particularly their family and friends. 

## 4. Perceived Religious Endorsement for Nursing

The life of a Muslim is guided by two major sources: the Holy Quran and the Sunnah. The Holy Quran is the book believed to convey the words of God relayed to Prophet Muhammad through the Angel Gabriel. The Sunnah refers to the deeds and sayings of the Prophet Muhammad, including everything he stated, reported, did, and described. Islam, as a religion, is very comprehensive, and it has specific instructions for almost every aspect of life, including nursing. Many good tidings mentioned in prophetic remarks about nursing and taking care of patients (known as Hadits) can be found in the Sunnah. Such narrations are mentioned below.

“Whoever visits a patient or visits his fellow brother, an angel says: you are blessed and your steps are blessed and you have made an abode in paradise.”

(Bukhari [15]) (p. 436)

“If a Muslim visits a sick Muslim in the morning seventy thousand angels invoke blessings on him till the evening; and if he visits in the evening, then seventy thousand angels invoke blessings on him till the morning, and for him, there is a garden of fruits in paradise.”

(Tirmidhi [15]) (p. 437)

“If a believer comforts his fellow brother in distress, Allah will clothe him with a dress of nobility on the Day of Reckoning.”

(Ibne Majah [15]) (p. 441)

The above narrations signify that looking after sick and distressed people is highly encouraged in Islam. Rufaidah bint Sa’ad, a woman from the Bani Aslam tribe in Medina who lived during the time of Prophet, is believed to be the first professional nurse in the history of Islam. Rufaidah learned the skill from her father, who was a physician by profession. In peacetime, she used to treat the patients in her camp just beside the mosque of the prophet. During times of war or conflict, she used to lead a team of volunteers to serve the injured soldiers [16]. The exemplary stories of Rufaida’s epic acts are still discussed among the Saudi nursing community and serve as the source of inspiration for today’s nurses [2]. However, there are also downsides of the profession that are counter to certain Islamic beliefs, particularly intermingling of men and women, which receives disapproval from Islamic leaders. El-Bar, a prominent critic of women working outside the home, has warned gravely about the likelihood of moral degradation due to the intermingling of genders [2,17]. Thus, a Muslim nurse with a strong belief in Islamic creeds may face a dilemma while serving as a professional nurse in an environment where interactions between men and women are frequent and almost unavoidable. His/her commitment to the career is likely to be diminished if he/she perceives a religious condemnation due to the choice of profession and certain practices it entails. 

## 5. Career Commitment

Career commitment can be viewed as one’s attitude toward one’s profession or vocation. It encompasses the dedication to work and career, setting personal career goals, and an identification with and involvement in those goals. In particular, it implies willingness and enthusiasm to work toward the chosen career (Blau, 1985 [18]). Career commitment determines the extent to which an individual is willing to engage in various work-related tasks, including those that extend beyond the immediate scope of his/her duties and the organizational goals [18]. Individuals committed to their career can see beyond the immediate work-related circumstances, as they strive toward a long-term goal. Thus, they are more likely to withstand pressure or other adverse circumstances, and tend to demonstrate greater dedication to their job and employer [19]. Conversely, career goals may prompt someone to change jobs multiple times, if those roles do not support their overall career path. This assertion is supported by the findings reported by Meyer, Allen, and Smith [20], who observed a moderate negative correlation between career commitment and intention to leave the profession among nurses. On the other hand, Gradner [21] and Somers [22] revealed that nurses with a lower level of occupational commitment were more willing to leave the profession, suggesting that the link between career commitment and dedication to a specific job or employer is complex and requires further study. Wang, Tao, Ellenbecker, and Liu [23] conducted similar research in the Chinese context, reporting a significant positive correlation between career commitment and nurses’ intent to stay. Numerous social, organizational, and demographic factors have been found to exert a moderating role on career commitment. Authors of extant research focusing on nurses have examined age [23,24], education [24,25], gender [26], marital status [24,27], tenure [24,25], level of organization [28], type of organization [29], among other factors. However, their findings are inconsistent, further confirming the need to explore antecedents and moderators of career commitment in greater detail. 

## 6. Research Question

Perceived social support and perceived religious endorsement as predictors of career commitment among nurses have not been explored in extant research. However, the role of social support in one’s ability to cope with stress has been investigated and was found to alleviate stress associated with emotionally sensitive diseases [30,31], depression [32], and work-family conflict [33], among others. These findings suggest that social support is likely to enhance career commitment among professionals whose job is associated with stigma and is deemed inappropriate from the societal, cultural, and religious perspective, as is the case for nursing in Saudi Arabia. Therefore, the aim of the present study was to investigate the effect of perceived social support (PSS) and perceived religious endorsement (PRS) on career commitment (CC) of Saudi nurses. As a part of the analysis, the effect of various demographic, social, and organizational factors that may influence the extent of PSS and CC, were also examined. Even though these variables have been extensively explored in the context of career commitment, research focusing on the role of demographic and other variables as moderators for perceived social support is limited. However, it is reasonable to assume that the level of PSS may vary depending upon the characteristics of the individuals and the nature of their job. For instance, to enter a stressful profession like nursing, young people should have a higher level of social support compared to their older counterparts. Similarly, owing to the male dominance and strict gender segregation in Saudi culture, it can be posited that female employees (nurses) will need more social support than their male counterparts. In addition, married employees are expected to require a higher level of social support relative to their unmarried colleagues, who do not need to balance home life and career aspirations. The authors further noted that advanced educational attainment in a particular field implies that the profession is endorsed by individual’s social network, family in particular. In addition, due to greater workload and job insecurity, private-sector employees may require a higher level of support and cooperation from their social network than needed by their public-sector counterparts. Guided by these assumptions, the current study focused on ascertaining the moderating effects (if any) of the key demographic and organizational factors on the level of perceived social support and career commitment among Saudi nurses. Therefore, the goal was to answer the following research question:

1. What is the effect of PSS and PRE on CC of Saudi nurses?
2. What are the moderating effects of a few selected demographic and organizational factors (age, gender, marital status, education, experience, and the type of organization) on the level of PSS and CC?

## 7. Materials and Methods

In this cross-sectional quantitative study, the data was collected by surveying Saudi nurses working in different government and private hospitals/clinics located in major cities of Saudi Arabia. As 146 of the 300 questionnaires distributed through professional research assistants were returned and thus available for analysis, a response rate of 49% was achieved. While questionnaires for perceived social support and career commitment were adopted from Zimet, Dahlem, Zimet, and Farley [34] and Saks [35], respectively, a new scale for perceived religious endorsement was developed specifically for the present study. The Multidimensional Scale of Perceived Social Support (MSPSS) developed by Zimet et al. [34] used to measure perceived social support includes 12 items, which cover three dimensions: Family, Friends, and Significant Others. Sample items include: “My family really tries to help me” (Family), “I can count on my friends when things go wrong” (Friends), and “There is a special person who is around when I am in need” (Significant Others). As noted above, career commitment was measured via the eight-item Affective Commitment Scale developed by Saks [35]. Sample items for commitment include: “I feel a strong sense of belongingness to my career” or “I would be happy to work in my profession until I retire.” In addition, a four-item scale was developed to measure the perceived religious endorsement of Saudi nurses, keeping the Islamic creeds in mind. The sample items include: “I feel encouragement from my religion to continue my profession as a nurse”. 

For all three variables (PSS, PRE, and CC), respondents were asked to rate the items on the scale from strongly disagree (1) to strongly agree (5), indicating 3 as the midpoint. Therefore, the higher the score, the higher the level of measured variable assumed. In addition to scale questions, the questionnaire also included queries related to demographic and organization specific information. The questionnaire was initially prepared in English. However, for easy comprehension, each question was translated into Arabic by an expert before being reviewed by two other experts to confirm the accuracy of the translation. Both English and Arabic versions of the questions were presented side-by-side in the final questionnaire. Multiple regression and independent sample *t*-test were used to analyze the survey data using Smart-PLS version 3 (SmartPLS GmbH, Hamburg, Germany) and SPSS version 22.0 (IBM Corporation, New York, NY, USA), respectively. 

### Ethical Considerations

In the absence of any committee or review board in the University to consider and approve the ethical aspects of this research, the authors took the initiative to ensure that all ethical considerations were adhered to. To maintain transparency, each questionnaire was accompanied by a cover letter, where it was explicitly mentioned that the participation was voluntary and that all individual responses would remain anonymous and confidential. The authors, therefore, strictly maintained the anonymity of the respondents and confidentiality of the data in all stages of the research.

## 8. Results

Table 1 represents the descriptive statistics pertaining to the study sample. As can be seen, the mean scores for all the variables are above 3, which indicates that the respondents mostly agreed with the questionnaire statements pertaining to perceived social support, career commitment, and perceived religious endorsement. Moreover, analysis of their demographic data indicates that most of the participants are relatively young, unmarried, diploma holders, work for government hospitals/clinics, and have at least five years or professional experience in the nursing field.

To conduct multiple regressions, we analyzed the data via Partial Least Squares (PLS) path model, using the Smart PLS 3 software. Following the guidelines given by Hair et al. [36], the results were interpreted in two stages: (1) assessment of the measurement model, and (2) evaluation of the structural model.

### 8.1. Measurement Model

**Figure 1:**
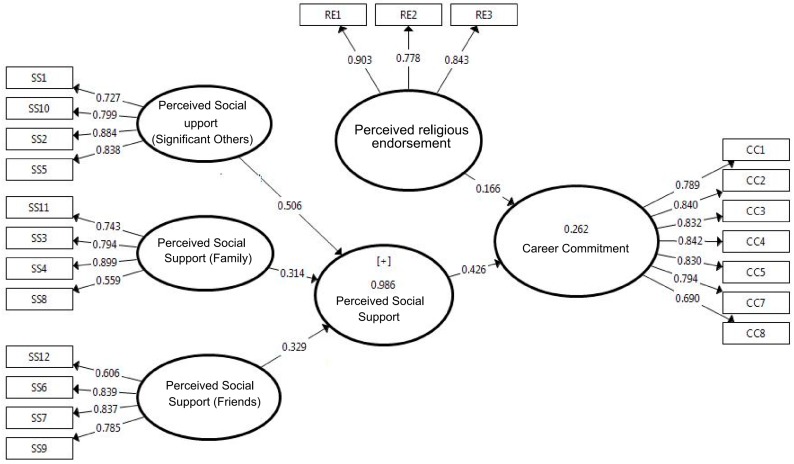
Factor loading of the model.

The results, as shown in Figure 1 and Table 2, indicate that the measurement models satisfy the minimum requirements. Factor loadings of most of the items related to perceived social support exceed 0.70, which confirms the indicators’ reliability. However, two indicators (SS8 and SS12) are observed to have lower loadings. The authors nevertheless decided to retain them because of the satisfactory levels of internal consistency, reliability, and convergent validity of the corresponding constructs. However, in case of perceived religious endorsement (PRE), the authors observed that one item (RE4) anchored with very low factor loading (0.148). Therefore, the authors deleted the item, which increased the overall Cronbach’s alpha from 0.507 to 0.794. Similarly, for career commitment, one item (CC6) with low factor loading (0.365) was removed, which considerably improved the scale reliability. 

With regard to composite reliabilities and Cronbach’s alpha, as all the constructs scored above 0.70 (Table 2), the measures’ internal consistency and reliability are supported. In addition, all average variance extracted (AVE) values exceed the threshold of 0.50, confirming the convergent validity of the measures. The analysis also validates discriminant validity with respect to Fornell-Larcker Criterion by comparing the square root of AVE to the correlations (Table 2).

### 8.2. Structural Model

The results yielded by the structural model were analyzed in line with the strategy adopted by Hair, Hult, Ringle, and Sarstedt [36]. According to the analysis findings, all the variance inflation factor (VIF) values are lower than the acceptable threshold of 5. Therefore, it can be deduced that each set of predictors in the structural model maintains minimum collinearity [37]. The value of R^2^ for the endogenous construct is 0.289, which is above the adequate level of 10% recommended by Falk and Miller [38]. However, according to the rules of thumb, this value is rather weak, as it implies that the Saudi nurses’ career commitment is affected by a multiplicity of factors. Likewise, results obtained through blindfolding with an omission distance of 7 yielded Q^2^ values that substantially exceed the recommended value of zero (Table 3). This finding confirms the model’s predictive relevance in terms of out-of-sample prediction [39].

**Figure 2:**
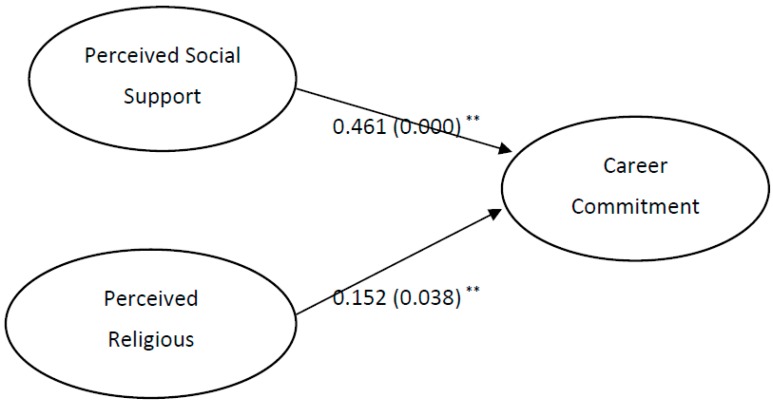
Structural model with path coefficients and *p*-value. (** Indicates significant at 5% level).

To calculate the *p*-values, along with the corresponding 95% bias-corrected and accelerated (BCa) bootstrap confidence intervals for the hypothetical paths, the bootstrapping procedure (performed on 5000 bootstrap samples with no sign changes) was applied and the results are presented in Figure 2. The *p*-values of both path coefficients indicate significant positive relationships between perceived social support and career commitment, as well as between perceived religious endorsement and career commitment. These findings indicate that career commitment of Saudi nurses is significantly affected by both perceived social support and perceived religious endorsement. However, the path coefficients indicate a stronger effect of PSS than PRE on career commitment. 

### 8.3. Independent Samples t-Test

Independent samples *t*-tests were performed in order to compare the mean scores of perceived social support and career commitment in relation to different demographic and organizational variables. As shown in Table 4, for most cases, no significant differences were observed. However, nurses working for private hospitals/clinics were found to exhibit a significantly higher career commitment compared to their counterparts working in government hospitals. Moreover, perceived social support and career commitment of nurses that hold a diploma and BSc degree were also significantly higher than among nurses who completed the certificate course only. 

## 9. Discussion

The goal of the present investigation was to explore the relationship that perceived social support and perceived religious endorsement have with career commitment of Saudi nurses. The findings revealed a strong positive impact of perceived social support and perceived religious endorsement on career commitment of the study participants. Given that nursing is not seen as a prestigious career in Saudi Arabia, and women are in general discouraged from working outside the home, the findings reported in this work support the widely held view that social support has a strong contribution to one’s ability to cope with job-related stress [11,12]. High level of perceived social support (4.02) indicates that the Saudi nurses receive considerable support from their family, friends, and other members of their social network to continue performing their job as a nurse. Moreover, very high score (4.57) measured for perceived religious endorsement implies that the Saudi nurses that took part in this study feel that their job is not in contradiction with their religion (Islam). Rather, they consider it as a pro-religious act, as helping others is a noble endeavor, which is highly encouraged in Islam. Indeed, since intermingling with the opposite gender is the major concern related to the health profession in general, the gender-based segregation of patients and facilities in most of the hospitals and clinics in Saudi Arabia may have contributed to this positive view of the nursing profession and the high level of perceived religious endorsement. In addition, as the majority of the nursing roles in Saudi Arabia are still predominantly performed by expatriates, the burden on Saudi nurses to undertake undesirable aspects of patient care may be lessened, as they are less likely to work night shifts or in intensive care units and are typically not required to handle sensitive patients or deal with the patients and their family members of the opposite sex. Therefore, the limited scope of their duties, skewed toward the more positive aspects of the role, may explain the relatively high level of perceived support from the participants’ social networks, as well as from the religious entities. The reluctance of Saudi nurses to undergo some of the more painful aspects of the job, such as night shifts or handling sensitive patients, can be attributed to the recently emerged work ethics in modern-day Saudi society. After the discovery of “black gold” (oil) in the early 1970s, the Kingdom experienced a huge influx of workers from abroad who, along with knowledgeable and skilled experts in specific fields, also took care of most of the menial jobs, such as cleaning, construction work, etc. Consequently, Saudi families gradually became dependent on expatriates for meeting their needs in all aspects of daily life. One glaring example is the number of domestic workers in Saudi Arabia, most of whom are not Saudi nationals. According to a news report, approximately 1.5 million housemaids are working in the Kingdom [40]. Such practices convey an undesirable aspect of the Saudi work culture, which holds some professions in extremely low esteem. Consequently, Saudi nationals performing such jobs are subjected to humiliation by the members of their tribe, clan, or even family [41]. Mohsin Shaikh Al-Hassan, host of a popular television program, “Jobs on Air,” in an interview blamed the role of family for not nurturing the right work ethics among the young generation. He maintained that Saudi families did not train their children at an early age to do chores at home; instead, they provide everything the child needs and typically employ foreign workers to perform the most menial tasks, which ultimately leads to inertia and disrespect toward certain service professions [42]. Thus, it is not unlikely to experience apathy among Saudi nurses to show the true sense of professionalism in their jobs.

The present study findings also revealed no significant differences in perceived social support and career commitment between male and female nurses. This is in line with the results reported by Morrow and Wirth [27], Lee, Carswell, & Allen [43], and Shafer et al. [26] and is attributed to the gender-based segregation in Saudi hospitals and clinics. Similarly, when the study sample was segregated into age groups, no differences emerged in either perceived social support or career commitment. Similar results were also noted by Colarelli and Bishop [44], Irving, Coleman, and Cooper [45], and Meyer, Stanley, Herscovitch, and Topolnytsky [46], among others. When marital status was examined as a potential moderator, no significant differences in the career commitment between married and unmarried nurses emerged, which is in line with the findings reported by Blau [18]. A high level of perceived social support reported by the nurses, regardless of their marital status, may have contributed to this result. Years of experience was yet another factor that showed little significance on the levels of perceived social support and career commitment. These observations are supported by the work of Cherniss [47] and Cohen [48], who reported no significant correlation between occupational loyalty and tenure. 

Private-sector nurses are found to have a significantly higher level of career commitment than their public-sector counterparts. While Mrayyan & Al-Faouri [49] reported similar results in the case of Jordanian nurses, the findings of this study contradict those reported by the same authors in another study [50]), as well as those of Lima et al. [29]. This discrepancy may be attributed to the fact that, even though the jobs in the private sector are more stressful, they tend to be financially more rewarding. Thus, despite recent government initiatives aimed at increasing the number of Saudi nurses in government hospitals and clinics, those who opt for nursing as a profession may still choose to join private sector in order to enjoy better financial and other fringe benefits relative to those available in public healthcare sector. The level of education was also shown to influence the level of perceived social support among the nurses that took part in the present study. Specifically, those who hold only a nursing certificate report significantly lower level of social support than do their colleagues that have Diploma and BSc qualifications. These differences may arise because the financial rewards, as well as job status, increase with the level of education the nurses possess. Usually nurses with certificate courses work as technical nurses, while the nurses with Diploma and BSc are given senior technical and specialist positions, respectively, with the corresponding level of power and respect, as well as greater financial benefits. As a result, close family, friends, and other members of the nurses’ social network may choose to overlook the negative aspects of the profession for better-qualified nurses and extend them greater support. Conversely, the duties performed by technical nurses (those who completed certificate courses only) are seen as more menial, and are perceived as “disgraceful” and thus they are rendered lower levels of social support. This finding implicitly hints at the tradeoff between the cultural values and material gains. It also may explain why nurses with a Diploma and BSc qualifications are more committed to their career than their less educated colleagues. Several authors [20,51] reported similar results, as they found a significant positive correlation between skill development or educational status and occupational commitment, possibly due to the greater time and effort investment into education by the more qualified nurses.

## 10. Conclusions

This pioneering study examined perceived social support and religious endorsement as the antecedents of career commitment in the context of Saudi nursing profession. It has revealed that both social support and religion play a vital role in determining the career commitment among the studied sample. Therefore, in order to enhance professionalism in the nursing sector in Saudi Arabia, greater efforts should be invested in improving public awareness of the importance and value of nursing for the national health and wellbeing. Moreover, religious discussions should emphasize compassionate and altruistic aspects of nursing and should promote greater support from family and broader social networks. Finally, to increase career commitment among Saudi nurses, the government should provide them with a greater level of responsibility and empowerment, along with better financial rewards. 

## 11. Limitations and Direction for Future Studies

This is a cross-sectional study where data were collected from a small non-probabilistic sample. Thus, the results should be studied with caution and cannot be generalized. As it is based on the self-administered survey, the study is susceptible to the limitations related to such a data collection method. To understand the cultural embeddedness of the Saudi nursing profession, more intensive studies are required to explore public attitude toward the profession and its connection to socio-cultural and religious factors. The present practice of gender-based segregation in Saudi hospitals and clinics also should be studied to investigate the view of Saudi nurses and other healthcare staffs as well as the general public and its relationship with the career commitment, job satisfaction, and other work-related variables of healthcare employees.

## Figures and Tables

**Table 1:** Sample descriptive.

Gender			Age	
Male	Female		Below 30 years	Above 30 years
57 (39%)	89 (61%)		112 (77%)	34 (23%)
Experience			Marital Status	
Below 5 Years	Above 5 Years		Single	Married
62 (42%)	84 (58%)		83 (57%)	63 (43%)
Education			Organization Type	
Certificate	Diploma	BSc.	Public	Private
17 (12%)	79 (54%)	50 (34%)	119 (81%)	27 (19%)
Scale Variables			Mean	Standard Deviation
Perceived Social Support (Significant Others) (PSSSO)			4.07	0.65
Perceived Social Support (Family) (PSSF)			4.06	0.61
Perceived Social Support (Friend) (PSSFR)			3.92	0.59
Overall Perceived Social Support (PSS)			4.02	0.53
Perceived Religious Endorsement (PRE)			4.57	0.53
Career Commitment (CC)			3.98	0.75

**Table 2:** Measurement model indicators.

Constructs	Cronbach’s Alpha	rho_A	Composite Reliability	Average Variance Extracted (AVE)	
Perceived Social Support-Family (PSSF)	0.740	0.768	0.841	0.575	
Perceived Social Support-Friends (PSSFR)	0.774	0.808	0.854	0.597	
Perceived Social Support-Significant Others (PSSSO)	0.828	0.836	0.886	0.662	
Perceived Social Support (PSS)	0.879	0.887	0.902	0.504	
Perceived Religious Endorsement (PRE)	0.794	0.804	0.880	0.710	
Career Commitment (CC)	0.908	0.915	0.927	0.646	
Discriminant Validly (Fornell-Larcker Criterion)					
	CC	PSSF	PRE	PSSFR	PSSSO
CC	0.804				
PSSF	0.470	0.759			
PRE	0.326	0.347	0.843		
PSSFR	0.380	0.552	0.304	0.773	
PSSSO	0.408	0.651	0.309	0.612	0.814

**Table 3:** Collinearity, R2 and Q2.

Factors	Collinearity (VIF)		
	CC	R^2^	Q^2^
CC		0.289	0168
PSS	1.166		
PRE	1.166		

**Table 4:** Independent sample *t*-test for mean scores of respondents on perceived social support (PSS) and career commitment (CC).

Scale	Gender				
	Male (N = 57)	Female (N = 89)	F	T (sig.)	Conclusion
PSS	4.04	3.99	0.40	0.57	No significant difference.
CC	3.92	4.03	1.17	0.37	No significant difference.
Organization Type					
	Govt. (N = 119)	Private (N = 27)	F	T (sig.)	
PSS	4.02	3.99	0.40	0.82	No significant difference
CC	3.91	4.33	2.53	0.01	Significant difference at 0.01 level
Age					
	Below 30 (N = 112)	Above 30 (N = 34)	F	T (sig.)	
PSS	4.00	4.02	0.06	0.90	No significant difference.
CC	4.04	3.81	1.43	0.12	No significant difference.
Experience					
	Less than 5 Years (N = 62)	More than 5 Years (N = 84)	F	T (sig.)	
PSS	4.02	4.01	0.23	0.92	No significant difference.
CC	4.10	3.90	2.30	0.12	No significant difference.
Marital Status					
	Single (N = 83)	Married (N = 63)	F	T (sig.)	
PSS	4.00	4.03	0.09	0.69	No significant difference.
CC	4.05	3.90	0.21	0.21	No significant difference.
Education					
	Certifct. (N = 17)	Diploma (N = 79)	F	T (sig.)	
PSS	3.75	4.04	0.20	0.05	Significant difference at 0.05 level
CC	3.66	3.99	0.01	0.10	Significant difference at 0.10
	Certifct. (N = 17)	BSc. (N = 50)	F	T (sig.)	
PSS	3.75	4.06	0.07	0.05	Significant difference at 0.05 level
CC	3.66	4.09	0.00	0.04	Significant difference at 0.05 level
	Diploma (N = 79)	BSc. (N = 50)	F	T (sig.)	
PSS	4.04	4.06	0.10	0.79	No significant difference.
CC	3.99	4.09	0.02	0.49	No significant difference.
